# Peritraumatic Distress, Watching Television, and Posttraumatic Stress Symptoms among Rescue Workers after the Great East Japan Earthquake

**DOI:** 10.1371/journal.pone.0035248

**Published:** 2012-04-25

**Authors:** Daisuke Nishi, Yuichi Koido, Naoki Nakaya, Toshimasa Sone, Hiroko Noguchi, Kei Hamazaki, Tomohito Hamazaki, Yutaka Matsuoka

**Affiliations:** 1 Department of Psychiatry, National Disaster Medical Center, Tachikawa, Japan; 2 Clinical Research Institute, National Disaster Medical Center, Tachikawa, Japan; 3 CREST, Japan Science and Technology Agency, Tachikawa, Japan; 4 Head Office, Japan Disaster Medical Assistance Team, Tachikawa, Japan; 5 Department of Nutrition and Dietetics, Faculty of Family and Consumer Science, Kamakura Women's University, Kamakura, Japan; 6 Department of Rehabilitation, Faculty of Health Science, Tohoku Fukushi University, Sendai, Japan; 7 Department of Adult Mental Health, National Institute of Mental Health, National Center of Neurology and Psychiatry, Kodaira, Japan; 8 Department of Public Health, Faculty of Medicine, University of Toyama, Toyama, Japan; 9 Department of Clinical Sciences, Institute of Natural Medicine, University of Toyama, Toyama, Japan; 10 Clinical Research Training and Consultation Program, Translational Medical Center, National Center of Neurology and Psychiatry, Kodaira, Japan; Wayne State University, United States of America

## Abstract

**Background:**

The Great East Japan Earthquake of March 11, 2001 left around 20,000 dead or missing. Previous studies showed that rescue workers, as well as survivors, of disasters are at high risk for posttraumatic stress disorder (PTSD). This study examined the predictive usefulness of the Peritraumatic Distress Inventory (PDI) among rescue workers of Disaster Medical Assistance Teams (DMATs) deployed during the acute disaster phase of the Great East Japan Earthquake.

**Methodology/Principal Findings:**

In this prospective observational study, the DMAT members recruited were assessed 1 month after the earthquake on the PDI and 4 months after the earthquake on the Impact of Event Scale-Revised to determine PTSD symptoms. The predictive value of the PDI at initial assessment for PTSD symptoms at the follow-up assessment was examined by univariate and multiple linear regression analysis. Of the 254 rescue workers who participated in the initial assessment, 173 completed the follow-up assessment. Univariate regression analysis revealed that PDI total score and most individual item scores predicted PTSD symptoms. In particular, high predictive values were seen for peritraumatic emotional distress such as losing control of emotions and being ashamed of emotional reactions. In multiple linear regression analysis, PDI total score was an independent predictor for PTSD symptoms after adjusting for covariates. As for covariates specifically, watching earthquake television news reports for more than 4 hours per day predicted PTSD symptoms.

**Conclusions/Significance:**

The PDI predicted PTSD symptoms in rescue workers after the Great East Japan Earthquake. Peritraumatic emotional distress appears to be an important factor to screen for individuals at risk for developing PTSD among medical rescue workers. In addition, watching television for extended period of time might require attention at a time of crisis.

## Introduction

The Great East Japan Earthquake and tsunami that occurred on March 11, 2011 devastated the northeastern coast of Japan, and left about 20,000 dead or missing. Rescue workers belonging to the national network of Disaster Medical Assistance Teams (DMATs), established by the Ministry of Health, Labour and Welfare of Japan, were dispatched to the disaster area. Previous studies have shown that rescue workers, as well as survivors, are at risk for developing mental disorders. For instance, 13.5% of medical care personnel sent to assist trauma victims of an airline crash developed posttraumatic stress disorder (PTSD) within 18 months [1]. Similarly, 16.7% of rescue workers deployed to the site of the September 11 terrorist attack in New York developed PTSD and 21.7% developed depression at 13 months [2]. PTSD can be associated not only with higher psychiatric comorbidity and physical illnesses [3], but also high healthcare costs [4]. Given that rescue workers will return to their workplaces in various regions after providing disaster relief efforts, it is very difficult for psychiatric professionals to conduct interviews with all of them, and therefore an appropriate screening tool for PTSD is needed in order for effective secondary preventive strategies to be provided to individuals at high risk.

In the pathogenesis of PTSD, fear memory becomes excessively consolidated [5] and is thought to be enhanced by peritraumatic distress, the psychological distress experienced at the time of and immediately after trauma. Peritraumatic distress is also thought to sensitize the neurobiological system [6]. A meta-analysis showed that peritraumatic distress is one of the strongest predictors for PTSD [7], and the Peritraumatic Distress Inventory (PDI), a scale for assesing peritraumatic distress, has been shown to be a predictor for PTSD in accident survivors [8]. However, it remains to be proven whether the PDI score in the immediate aftermath of a disaster can predict for PTSD in rescue workers. To this end, this study examined the predictive usefulness of the PDI among DMAT members who were deployed during the acute disaster response phase of the Great East Japan Earthquake.

## Methods

### Participants

DMATs are dispatched as mobile, specialized medical teams that provide medical aid during the acute phase of a large-scale disaster (i.e. within around 48 hours). Following the Great East Japan Earthquake, DMAT activities commenced on the same day and concluded 11 days later on March 22. DMAT members (physicians, nurses, and operational coordination staff) deployed to the disaster area who were recruited to this study met the following inclusion criteria: 1) aged 18 years or older; 2) a native Japanese speaker or non-native speaker with Japanese conversational abilities; and 3) physically and psychologically capable of understanding and providing consent for study participation.

### Procedure

This study was conducted as part of the study named “Attenuating posttraumatic distress with omega-3 polyunsaturated fatty acids among disaster medical assistance team members after the Great East Japan Earthquake (APOP)”. The detailed study procedures have been reported elsewhere [9]. Briefly, a written guide to the APOP study was posted to the Emergency Medical Information System website by the DMAT office, and affiliated hospitals with DMAT members were notified of the posting by their local municipalities. All study documents were then mailed to DMAT members who had been deployed to the disaster area. Participants returned written informed consent forms to the DMAT office by fax or email. Participants in any separate trial were excluded in the present observational study.

In the baseline assessment conducted at 1 month after the earthquake, which has been reported in detail elsewhere [9], participants were surveyed about the following variables that were identified in previous research to be risk factors for PTSD [1], [10]: period of deployment, stress prior to deployment, injury during deployment, experience of saving a child during deployment, experience of contact with corpses, concern over radiation, and duration of time spent watching earthquake television (TV) news reports. The PDI, which was included in the mailed study documents, was completed at this time. The instrument is a 13-item self-report questionnaire measuring distress experienced during and immediately after a critical incident (total score range, 0–52) [11]. The response format is a five-point Likert scale that ranges from 0 to 4 (0 = not at all, 1 = slightly true, 2 = somewhat true, 3 = very true and 4 = extremely true). It typically takes just several minutes to complete all items. The Japanese version that we developed in cooperation with the original developers has been demonstrated to have good internal consistency, concurrent validity, and test–retest reliability [12]. A series of activities such as seeing frightful spectacle and listening to traumatized people were selected as a critical incident in the present study.

The primary outcome was total score on the Impact of Event Scale-Revised (IES-R) at 4 months after the earthquake. The IES-R is a self-reporting questionnaire about PTSD symptoms comprised of 22 items on the three most common symptoms in the diagnostic criteria of PTSD, namely re-experiencing, avoidance, and hyperarousal. It is the most widely used measure internationally in all forms of disaster-area research [13]. Respondents rate symptoms experienced in the previous week. The validity and reliability of the Japanese version of the IES-R has been confirmed [14].

### Ethics

The study protects the rights and welfare of participants in the spirit of ethical guidelines outlined under the Declaration of Helsinki, and further respects the ethical principles of the Ministry of Health, Labour, and Welfare of Japan. The study was approved by the Ethics Committee of the NDMC on April 1, 2011. Individual participants in this study provided written informed consent.

### Statistical analysis

Univariate regression analysis was used to examine the relationship of PDI total score and of PDI individual item scores with posttraumatic stress symptoms. In a model for determining the predictive value of PDI, multiple linear regression analysis was used to examine the relationship of the PDI with PTSD symptoms adjusted for the following covariates: age, being female, and history of psychiatric illness, which are well-established pretraumatic risk factors across trauma type [7], [15], and the other above-mentioned variables that were previously identified as risk factors for PTSD after disasters [1], [10]. Variables for which there were fewer than 5 respondents were excluded from the multiple linear regression analysis. Moreover, the relationships of the PDI with covariates were examined by calculating Pearson's correlation coefficients, or using t-test, or using analysis of variance. The relationships of covariates with PTSD symptoms were also examined by univariate regression analysis.

Any association between the dependent variable and the independent variable was expressed as a regression coefficient (beta weight) and quantified by the 95% confidence interval (95% CI). All statistical analysis used two-tailed tests. Statistical significance was established at a P value of less than 0.05. All data analysis was performed using SPSS statistical software version 19.0J for Windows (SPSS, Tokyo, Japan).

### Results

**Figure 1:**
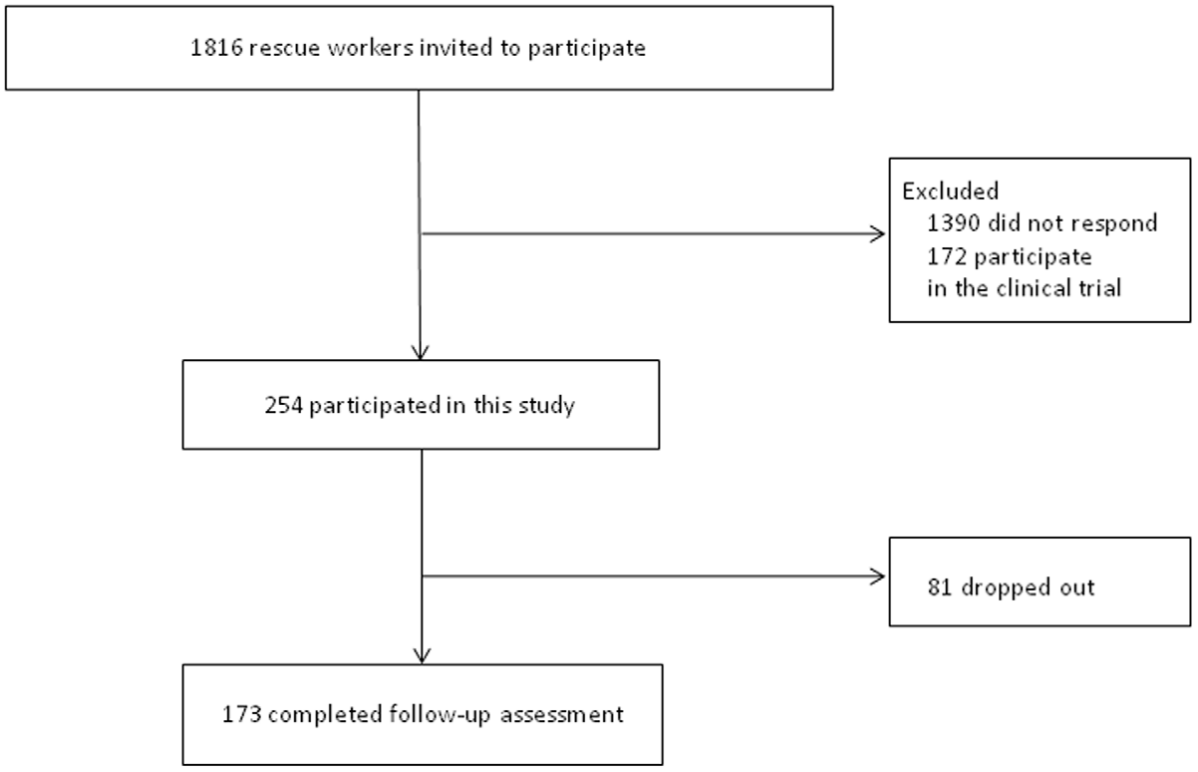
Flow diagram of the study.

Of the 1,816 DMAT workers deployed to the disaster areas, 172 participated in another intervention trial and 1390 did not respond. Thus, 254 participants were recruited to this observational study and provided baseline data collected during the period April 2 and 22, 2011 (Figure 1). Of these 254 participants, 173 (68.1%) completed the follow-up assessment at 4 months after the earthquake, with data collected between July 11 and August 4, 2011. The participants who dropped out of the study were more likely to be men (p = 0.04), not to have been stressed prior to deployment (p = 0.047), and with experience of saving a child during the deployment (p = 0.02). Otherwise there were no significant differences in variables including PDI total score between study completers and non-completers. Demographic and exposure characteristics of the 173 completers are shown in Table 1. Most participants were not exposed to saving a child or contact with corpses, and none was injured. The mean duration from baseline assessment to follow-up assessment was 98.2 days (SD 5.4). Of the 75 women participants, 65 (86.7%) were nurses.

**Table 1:** Demographic and exposure characteristics of rescue workers who participated in the follow-up study.

Variables	n	%	Mean	SD	Median	Range
Age			38.8	7.6		
Sex, women	75	43.4				
History of psychiatric illness, yes	4	2.3				
Occupation						
Doctors	35	20.2				
Nurses	80	46.2				
Others	58	33.5				
Period of deployment (days)					3.7	1–12
Stress prior to deployment, yes	53	30.6				
Injury during deployment, yes	0	0				
Saving a child during deployment, yes	4	2.3				
Experience of contact with corpses, yes	14	8.1				
Concern over radiation, yes	13	7.5				
Watching earth quake news reports						
<1 hour	42	24.3				
1–4 hours	119	68.8				
≥4 hours	12	6.9				
Baseline peritraumatic distress (PDI) score			13.2	7.5		
IES-R score at 4 months after the earthquake			6.8	8.4		

PDI, Peritraumatic Distress Inventory; IES-R, Impact of Event Scale-Revised; SD, standard deviation.

The results of univariate and multiple linear regression analysis were shown in Tables 2 and 3, respectively. History of psychiatric illness, injury during deployment, and saving a child during deployment for which there were fewer than 5 respondents were excluded from the multiple linear regression analysis. PDI total score and most of the individual item scores predicted PTSD symptoms in the univariate regression analysis. Moreover, PDI total score was an independent predictor for PTSD symptoms after adjusting for the covariates (beta = 0.43, 95% CI, 0.27–0.59; p<0.01). R square values for the multiple linear regression model was 0.30. Among the covariates, watching TV for more than 4 hours per day at 1 month after the earthquake was predictive of PTSD symptoms. Values of variance inflation factor did not exceed 1.4, which indicated that multicollinearity did not seem to be an issue. PDI total scores were significantly higher in participants participants watching TV for more than 4 hours per day (19.6) than for 1 to 4 hours (12.7) and for less than 1 hour (12.6), but no other significant associations were seen between PDI and other covariates.

**Table 2:** Results of univariate regression analysis.

Item description	*Beta* (95% *CI*)	*R* square	*p* value
1. I felt helpless to do more	2.76 (1.57, 3.95)	0.11	<0.01
2. I felt sadness and grief	1.69 (0.66, 2.73)	0.06	<0.01
3. I felt frustrated or angry I could not do more	2.14 (1.15, 3.13)	0.10	<0.01
4. I felt afraid for my safety	1.62 (0.48, 2.76)	0.04	0.01
5. I felt guilt that more was not done	2.39 (1.41, 3.37)	0.12	<0.01
6. I felt ashamed of my emotional reactions	4.00 (2.63, 5.38)	0.16	<0.01
7. I felt worried about the safety of others	1.65 (0.65, 2.65)	0.06	<0.01
8. I had the feeling I was about to lose control of my emotions	4.81 (3.43, 6.20)	0.22	<0.01
9. I had difficulty controlling my bowel and bladder	−0.71 (−4.75, 3.34)	0.00	0.73
10. I was horrified by what happened	1.12 (0.28, 2.10)	0.04	0.01
11. I had physical reactions like sweating, shaking, and pounding heart	3.48 (2.09, 4.87)	0.13	<0.01
12. I felt I might pass out	4.99 (0.94, 9.04)	0.03	0.02
13. I felt I might die	2.96 (1.66, 4.23)	0.10	<0.01
Total	0.53 (0.38, 0.68)	0.23	<0.01

*CI*, confidential interval.

*R* ^2^, multiple correlation coefficient, index of goodness fitness in the model.

**Table 3:** Results of univariate and multiple linear regression analysis.

	Univariate regression*Beta* (95% *CI*)	Mulitiple linear regression*Beta* (95% *CI*)
PDI per 1 point	0.53 (0.38, 0.68)**	0.43 (0.27, 0.59)**
Covariates		
Age	−0.18 (−0.34, 0.01)*	−0.07 (−0.22, 0.08)
Sex, women	4.95 (2.51, 7.39)**	2.11 (−0.24, 4.47)
period of deployment	−0.25 (−1.23, 0.73)	−0.18 (−1.03, 0.68)
stress prior to deployment	0.47 (−2.28, 3.21)	−0.07 (−2.48, 2.35)
experience of contact with corpses	3.63 (−0.98, 8.24)	2.87 (−1.20, 6.93)
concern over radiation	7.44 (2.77, 12.1)**	2.71 (−1.66, 7.07)
watching earthquake news report^a^		
<1 hour per day	Reference	Reference
1–4 hours per day	1.73 (−1.14, 4.61)	1.20 (−1.40, 3.80)
≥4 hours per day	10.3 (5.02, 15.5)**	5.24 (0.27, 10.2)*

*CI*, confidential interval.

PDI, Peritraumatic Distress Inventory.

a Entered as for <1 hour per day, 1 for 1–4 hours, and 2 for ≥4 hours.

* ,*p*<0.05;

** ,*p*<0.01.

## Discussion

This study showed that the PDI could predict PTSD symptoms in rescue workers at 4 months after the earthquake, even when adjusted for covariates in a multiple linear regression analysis. The predictive value of the PDI (beta 0.43) was compatible with that found for accident survivors (beta 0.49) [8]. The advantages of the PDI are that it can be completed quickly as well as immediately after traumatic events and by rescue workers themselves. It could therefore serve as a useful screening tool for the early identification of rescue workers at risk for developing PTSD.

Interestingly, items “I had the feeling I was about to lose control of my emotions” and “I felt ashamed of my emotional reactions” on the PDI showed higher predictive values for posttraumatic stress symptoms. We previously reported that items “I felt helpless to do more” and “I had physical reactions like sweating, shaking, and pounding heart” showed higher predictive values for PTSD symptoms than the other items among accident survivors, and neither item “I had the feeling I was about to lose control of my emotions” nor “I felt ashamed of my emotional reactions” predicted PTSD symptoms significantly [16]. This difference in findings could provide insights into the characteristics of rescue workers. Rescue workers have expectations and can prepare themselves for their experiences in the disaster area before arriving at the disaster area and may then have experiences that are totally different from their initial expectations. Therefore, once they are overwhelmed by those experiences, their psychological shock may be stronger than lay people. Accident survivors, on the other hand, have no prior warning and are mostly preoccupied by their own pain or sense of life threat following a sudden traumatic event. Loss of emotional control is not likely to cause a serious problem for them. This may be one reason why emotional distress is such an important factor to consider in the case of rescue workers. The importance of peritraumatic emotional distress, as conveyed by items such as “I had the feeling I was about to lose control of my emotions” or “I felt ashamed of my emotional reactions”, may need to be emphasized in the screening of individuals at high risk for developing PTSD among medical rescue workers.

Only item “I had difficulty controlling my bowel and bladder” on the PDI did not predict PTSD symptoms at all. This is consistent with our findings in accident survivors [16]. Previous studies, too, among participants in the United States, France, and Japan showed that this item was least endorsed [11], [12], [17]. Further studies are needed in other types of trauma, but this item may not be necessary to include in the future.

PDI was designed to assess the diagnostic criterion A2 of the PTSD, which required fear, helplessness, or horror at the time of the event [18]. At this time, removal of criterion A2 has been proposed in DSM-5 as it had no utility [19]. Indeed, as literatures and our previous study showed, the presence of peritraumatic distress is known as a weak indicator of the presence of PTSD [8], [20], [21]. However, the absence of peritraumatic distress is known as a strong indicator of the absence of PTSD [8], [21]. In addition, the time of assessment should be emphasized to minimize the effects of inaccurate memory over time. The present study suggested that A-2 criterion and PDI seemed to be still useful, at least in settings where peritraumatic distress could be assessed just after the events. Further studies are needed to elucidate what kinds of peritraumatic distress would be important for various traumatic events.

As for the covariates, we found a significant association between watching TV for more than 4 hours per day and PTSD symptoms after controlling for other covariates and PDI. Although the number of participants watching TV for more than 4 hours was only 12 (Table 1), our findings about the association between watching TV and PTSD symptoms were consistent with previous studies [10], [22], [23]. The associations may be explained by two ways. First, watching TV might be traumatic exposure. Generally, watching TV has not been regarded as an exposure to a traumatic event [18]. However, the study following the terrorist attacks of September 11 showed that those who watched TV frequently were more likely to have PTSD than those who did not among people who were directly affected by the attacks [22]. Because all participants in the present study were directly affected by the disaster, they might be affected by watching TV more than general population. Second, as Ahern et al. pointed out [23], watching TV for extended period of time might be a part of ineffective coping. Silver et al. showed that association between watching TV and PTSD symptoms was reduced after adjustment for coping strategies, especially active coping [24]. In this study, PDI total score was significantly higher in participants watching TV for more than 4 hours. This study did not show whether those with higher PDI score watched more TV as a part of ineffective coping or whether those watching more TV reported higher PDI score. Genetic and epigenetic factors might have made subjects vulnerable to both peritraumatic distress and ineffective coping, which needs further research.

In conclusion, PDI predicted PTSD symptoms in rescue workers after the Great East Japan Earthquake. In particular, factors concerning peritraumatic emotional distress, such as losing control of the emotions and being ashamed of emotional reactions, appear to be important in the screening of medical rescue workers. In addition, it would be desirable that time spent watching TV in the aftermath of a disaster is limited to a certain level. Rescue workers who have to watch a lot of TV in their work should be aware that it could associate with more posttraumatic stress symptoms.

### Limitations

First, as shown in Figure 1, 1,390 DMAT members who were invited to participate in the study did not respond, which could limit the external validity of the findings. This might be because many rescue workers dedicated themselves to continuing their important work at their own hospitals immediately after returning from their deployment and could not find the time to participate in this study. It remains unclear whether similar findings would have been obtained if the full cohort had been recruited successfully. There is a possibility that subjects whose mental status was not severely affected by the experiences during rescue activity might not have participated in the present study because of lack of motivation in general. Second, the attrition rate was relatively high. Because men and those who were not stressed prior to deployment were likely to drop out, more PTSD symptoms may have been reported if the attrition rate was much lower. Third, self-reporting questionnaires were used to determine the study outcomes for PTSD symptoms. Because PTSD could not be diagnosed by the IES-R, a cut-off score for the rescue workers remains unknown and we could therefore not analyze sensitivity and specificity. However, the IES-R seems to be a reasonable assessment method given this type of emergency situation.
